# Firework-related ocular trauma in Pernambuco, Brazil

**DOI:** 10.5935/0004-2749.2022-0205

**Published:** 2023-03-20

**Authors:** Tiago Cavalcanti de Carvalho, Rodrigo Pessoa Cavalcanti Lira, Caio Rodrigo de Oliveira Melo, Ana Karine de Araújo Soares, Camilla da Silva Rocha, Camila V. Ventura

**Affiliations:** 1 Department of Ophthalmology, Fundação Altino Ventura, Recife, PE, Brazil; 2 Department of Ophthalmology, Universidade Federal de Pernambuco, Recife, PE, Brazil; 3 Cornea and External Disease Department, Fundação Altino Ventura, Recife, PE, Brazil; 4 Scientific Research Department, Fundação Altino Ventura, Recife, PE, Brazil

**Keywords:** Emergencies, Eye burns/epidemiology, Fires, Blast injuries, Explosive agents, Emergências, Queimaduras oculares/epidemiologia, Incêndios, Traumatismos por explosões, Substâncias explosivas

## Abstract

**Purpose:**

This study aimed to describe the demographic and clinical characteristics of victims of fireworkrelated ocular trauma treated at the ophthalmologic emergency de partments of two reference centers in Pernambuco, Brazil, and to identify risk factors related to poor visual prognosis.

**Methods:**

We retrospectively evaluated the medical records of patients admitted in emergency departments with a report of firework-related trauma between January 2012 and December 2018. Data collected included patient’s age, sex, place of origin, month and year of the accident, ocular structures affected, characteristics of the injuries, and type of treatment that patients received. For patients who were followed for >30 days, the final visual acuity and patient’s origin were analyzed.

**Results:**

Three hundred and seventy eyes from 314 patients were included, of which 248 (79.0%) were male and 160 (51.0%) were from the metropolitan region of Recife. The mean patient age was 25.6 ± 18.8 years. In 56 (17.8%) patients, the ocular trauma was bilateral. A total of 152 (48.4%) cases occurred in June. The most affected sites were the eyelids in 91 (24.6%) eyes and ocular surface in 252 (68.1%). Surgical treatment was required in 87 (23.5%) eyes. After clinical and surgical management, 37 (10.0%) eyes presented final visual acuity of <20/400. Of these, 34 (91.9%) eyes were from patients from the countryside or from another state. Patients from the countryside presented higher risk of developing blindness after a firework trauma than those from the metropolitan area (odds ratio of 5.46).

**Conclusions:**

Victims of firework-related ocular trauma were mostly male, from the metropolitan region of Pernambuco state and mainly pediatric patients or economically active. Those coming from the countryside and other states had higher risk of developing blindness

## Abstract (pt)

**Objetivos:**

Descrever as características demográficas e clínicas das vítimas de trauma ocular por fogos de artifício atendidas nas emergências oftalmológicas de dois centros de referência em Pernambuco e identificar fatores relacionados a mau prognóstico visual.

**Métodos:**

Avaliação retrospectiva dos prontuários de pacientes admitidos na emergência oftalmológica com história de trauma por fogos de artifício entre janeiro de 2012 e dezembro de 2018. A coleta de dados incluiu idade, gênero, procedência, mês e ano do acidente, estruturas oculares acometidas e características das lesões, além do tipo de tratamento a que os pacientes foram submetidos. Naqueles pacientes acompanhados por mais de 30 dias, analisou-se a acuidade visual final e a associação com sua procedência.

**Resultados:**

Foram incluídos 370 olhos de 314 pacientes. Destes, 248 (79,0%) vítimas eram do sexo masculino e 160 (51,0%) da região metropolitana do Recife, com uma média de idade de 25.6 ± 18.8 anos. Em 56 (17,8%) dos casos o trauma foi bilateral. No mês de junho ocorreu um total de 152 (48,4%) casos. Os sítios mais acometidos foram pálpebras em 91 (24,6%) olhos e superfície ocular em 252 (68,1%). O tratamento cirúrgico foi necessário em 87 (23,5%) olhos. Após manejo clínico-cirúrgico, 37 (10.0%) olhos desenvolveram visão pior do que 20/400. Destes, 34 (91,9%) olhos eram de pacientes do interior do estado de Pernambuco ou de outro estado. Os pacientes provenientes do interior do estado apresentaram maior chance de desenvolver cegueira quando comparados aos que eram provenientes da região metropolitana (Odds Ratio de 5,46).

**Conclusões:**

As vítimas de trauma ocular por fogos de artificio foram em sua maioria do sexo masculino, procedentes da região metropolitana do estado e das faixas etárias pediátrica e economicamente ativa. Aqueles provenientes do interior ou de outros estados apresentaram maior chance de desenvolver cegueira.

## INTRODUCTION

Fireworks have an important historical, cultural, and religious meaning^(1)^, but they are also a known cause of serious accidents^(2,3,4,5,6,7,8,9)^. They are widely used for entertainment and celebrations in many countries but can bring irreversible damage to the health of those who run them or simply watch the show^(8)^.

In the USA, more than 97,500 patients presented to the emergency department because of firework-related injuries between 2000 and 2010, and approximately 82.0% of the cases involved the hands, eyes, face, or head^(10)^. Approximately 85,800 firework-related lesions were treated in pediatric patients between 1990 and 2003 in North-American emergency departments, and the ocular globe was the most frequently affected region, accounting for 20.8% of the cases^(11)^.

Eye traumas are a cause of morbidity, visual impairment, and socioeconomic losses; however, the absence of standardized registries can make its comprehension, monitoring, and prevention more difficult worldwide because its epidemiology is not widely known in many regions^(12)^.

A multicenter study of 388 patients showed severe vision loss in 18.1% of firework-related eye traumas, after a 4-week follow-up, which occurred during festive celebrations in several countries such as India, Nepal, Argentina, and the Netherlands^(13)^. In Brazil, no registration system is specific for ocular trauma; however, information on this condition can be found in some regional studies^(14,15,16,17,18)^.

Despite scarce information regarding firework-related ocular injuries in Brazil, they were pointed as the cause of 3.7% of serious ocular injuries in 216 patients in a university hospital in São Paulo^(18)^. This study evaluated patients treated for firework-related ocular trauma in two referral centers in the state of Pernambuco, Brazil, to investigate and identify possible factors that influenced the poor prognosis of these injuries.

## METHODS

A retrospective analysis was performed on patients with firework-related ocular trauma who attended the emergency departments of the Fundação Altino Ventura (FAV) and the Hospital de Olhos de Pernambuco (HOPE), public and private referral centers, respectively, for ophthalmology in Pernambuco, a Northeastern state in Brazil.

Medical records were used for patient selection and data collection. Data were collected from the charts of patients who had firework-related ocular trauma and attended the ophthalmological emergency departments of the FAV and HOPE between January 2012 and December 2018.

Sociodemographic data, clinical ophthalmologic data, and trauma-related data were analyzed. For the classification of mechanical injuries affecting the eyeball, the Birmingham Eye Trauma Terminology System^(19)^ was used. Corneal abrasions were classified as burns or contusions depending on other features described in the ophthalmologic examination, such as singed lashes or periocular abrasions. Full- or partial-thickness lid tears and partial (lamellar) lesions of the ocular wall were classified as lacerations. All data were categorized by the same examiner.

In cases with >30 days of follow-up, the final visual acuity of the injured eye was noted. Visual acuity was measured using a Snellen chart^(20)^ and registered as fractions. For visual acuities worse than 20/400, patients were evaluated for the ability to count fingers, see hand movements, or the presence or absence of light perception. For the categorization based on the visual acuity, the classification adopted by the *Conselho Brasileiro de Oftalmologia* and the World Health Organization was considered^(21)^.

Statistical analysis was performed using R version 3.2.3. Descriptive data were expressed as frequencies, average values, and standard deviations. The Chi-squared and Fisher’s tests were used for the evaluation of statistical significance. Odds ratios were calculated for estimating relative risk. A p-value <0.05 was considered statistically significant.

## RESULTS

This study analyzed 370 eyes of 314 patients who were admitted for firework-related ocular trauma in the two eye hospitals (FAV, n=290; HOPE, n=24). Male patients accounted for 248 (79.0%) cases. The mean patient age on admission was 25.6 ± 18.8 (range, 1.0-87.0) years, and 277 (88.2%) patients were <50 years old when the trauma occurred (Table 1).

**Table 1:** Patients’ distribution considering sex and age group

Age group (years)	Male n (%)	Female n (%)	Total n (%)
**0–**9	51 (16.2)	18 (5.7)	69 (22.0)
**10–19**	61 (19.4)	13 (4.1)	74 (23.6)
**20–29**	36 (11.5)	12 (3.8)	48 (15.3)
**30–39**	40 (12.7)	11 (3.5)	51 (16.2)
**40–49**	29 (9.2)	6 (1.9)	35 (11.1)
**50–59**	12 (3.8)	3 (0.9)	15 (4.8)
**60–69**	15 (4.8)	1 (0.3)	16 (5.1)
**70–79**	3 (0.9)	0 (0.0)	3 (0.9)
**80–89**	1 (0.3)	2 (0.6)	3 (0.9)
**Total**	**248 (79.0)**	**66 (21.0)**	**314 (100.0)**

**Figure 1:**
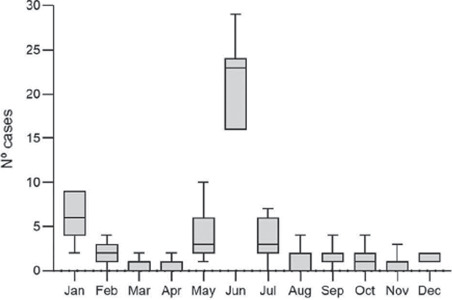
Distribution of firework-related ocular trauma over the months between 2012 and 2018.

June presented the highest incidence, accounting for 152 (48.4%) of the cases; however, January, May, and July also presented a high number of cases (Figure 1).

**Figure 2:**
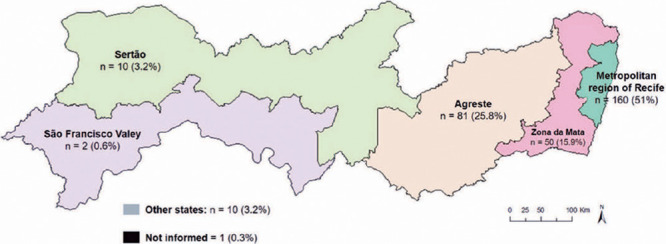
Distribution of cases according to patients’ origin in the mesoregions of the state of Pernambuco.

The left eye was injured in 136 (43.3%) patients, the right eye in 122 (38.9%) patients, and both eyes in 56 (17.8%). More than half of the patients were from the metropolitan region of Recife (Figure 2).

The most frequently damaged ocular structures were as follow: the ocular surface in 252 (68.1%) eyes, lids in 91 (24.6%), and anterior segment in 47 (12.7%). Posterior segment trauma was described in 44 (11.9%) eyes and orbit lesions in only 1 (0.3%). In those cases, multiple structures were damaged, except for one patient who presented with anterior uveitis, vitreous hemorrhage, and retinal detachment after a blunt trauma with no sign of ocular surface or other structural lesions. Two cases of endophthalmitis were detected, which occurred after open globe trauma and progressed to no light perception.

The main mechanisms of ocular trauma were burns, contusions, and superficial foreign bodies (Table 2). In 113 (30.5%) cases, more than one trauma mechanism was involved in the eye injury. Clinical management was employed in 283 (76.5%) eyes, whereas 87 (23.5%) had surgical intervention. Globe evisceration was performed in eight eyes, corresponding to 10.4% of patients who underwent eye surgery.

**Table 2:** Mechanisms of trauma and frequencies considering the total number of eyes injured

Single mechanism	n (%)	Associated mechanisms	n (%)
**Burn**	115 (44.7)	**Contusion + Burn**	47 (41.6)
**Contusion**	63 (24.5)	**FB + burn**	27 (23.9)
**FB**	56 (21.8)	**Contusion + Laceration**	15 (13.3)
**Rupture**	10 (3.9)	**FB + Contusion**	11 (9.7)
**Penetrating**	9 (3.5)	**Laceration + Rupture**	4 (3.5)
**IOFB**	2 (0.8)	**FB + IOFB**	1 (0.9)

FB= foreign body; IOFB= intraocular foreign body.

The final visual acuity was recorded in only 74 (20.0%) eyes (Table 3). Severe visual impairment (category 2) and blindness (categories 3-5) after trauma were identified in 42 (11.3%) eyes. Thirty-six patients developed blindness, of which 33 (91.7%) were male. One patient (2.8%) had bilateral trauma and developed bilateral blindness. Of the 37 eyes that developed blindness, 34 (91.9%) were eyes of patients from outside the metropolitan region of Recife (Agreste, Zona da Mata, Sertão, and São Francisco Valey) or from other states. Considering only patients from Pernambuco state who had their final visual acuity registered on medical charts, those from outside the metropolitan region had higher risk of developing blindness [odds ratio, 5.46 (p=0.009, CI = 1.38-21.49)] (Table 4). For the odds ratio analysis, cases were divided in groups according to their origin (countryside versus metropolitan region) and blindness outcome (yes versus no).

**Table 3:** Categories of visual deficiency and distribution of cases considering final visual acuity

Category	VA	n	%
**0**	**>20/60**	**25**	**33.8**
**1**	**<20/60 and** ≥**20/200**	**7**	**9.5**
**2**	**<20/200 and** ≥**20/400**	**5**	**6.7**
**3**	**<20/400 and** ≥**CF at 1 Meter**	**4**	**5.4**
**4**	**<CF at 1 Meter and** ≥**LP**	**16**	**21.6**
**5**	**NPL**	**17**	**23.0**
**Total**		**74**	**100.0**

CF= counting fingers; LP= light perception; NPL= no perception of light; VA= visual acuity. Categories: 0= mild or absence of visual deficiency; 1= moderate visual deficiency; 2= severe visual deficiency; 3-5= blindness.

**Table 4:** Distribution of cases according to the place of origin and visual deficiency categories considering the visual acuity on the last visit

		Categories of visual deficiency						
		0	1	2	3	4	5	Total
**Origin**	*Agreste*	11	4	3	3	6	5	32
	*Metropolitan region*	10	1	2	0	2	1	16
	*Sertão*	0	0	0	0	0	5	5
	*São Francisco Valey*	0	2	0	0	0	1	3
	*Zona da Mata*	3	0	0	1	5	3	12
	*Other state*	1	0	0	0	3	2	6
	**Total**	**25**	**7**	**5**	**4**	**16**	**17**	**74**

Categories: 0= mild or absence of visual deficiency; 1= moderate visual deficiency; 2= severe visual deficiency; 3-5= blindness.

## DISCUSSION

Fireworks are culturally used worldwide during celebrations and festivities. However, they are directly related to accidents and ocular injuries^(8)^. Studies have shown that approximately 75.0% of the patients are male, and when considering only severe trauma, this incidence is even higher in the male population reaching 96.7% of cases^(8,22)^. Hoskin et al.^(13)^ showed that the mean age of victims of firework-related trauma was 20.6 years. In the present study, both sexes and mean age of the victims corroborate with the results of previous studies^(6,22,23,24)^. Cultural aspects play an important role when explaining this predominantly male and young profile in firework-related ocular trauma. In general, men are more exposed to high-risk activities and trauma^(7)^.

Ocular trauma is an important ophthalmological emergency in Brazil^(14,17)^. At the FAV, in the period between January and June 2013, 5073 (19.3%) ocular trauma cases were seen^(14)^, from which 41 (0.8%) were firework-related traumas.

A firework explosion can cause ocular injuries such as hyphaema, vitreous hemorrhage, ocular wall laceration, retinal detachment, intraocular foreign bodies (IOFBs), and endophthalmitis^(22)^. The ocular surface and lids were among the most affected structures in other retrospective studies^(2,6,23)^. Studies have shown a prevalence of IOFB ranging from 2.7% to 13.0%^(6,22,24)^, which was higher than the prevalence in our population (0.8%). Regarding endophthalmitis, Jing et al.^(24)^ reported endophthalmitis in 3 of 25 (12%) eyes, and Kong et al.^(22)^ reported 6 in 118 (5.1%) eyes. Contrastingly, in our study, only 2 in 370 (0.5%) eyes had endophthalmitis.

In the present study, the main mechanism of injury in firework-related trauma was ocular burns. Similarly, a prospective evaluation during the Aidil Fitri celebration in 2008 in Malaysia showed that thermal mechanism comprised 60.0% of firework-related injuries^(25)^. Moreover, surgical intervention for firework-related ocular trauma is needed in 23.9%^(26)^-28.0%^(6)^ of cases, which was also similar to our data (23.5%). However, in a reference center in Northern China, this proportion reached 90.7% of cases, which was justified by the trauma profile and power of fireworks available in that region^(22)^.

In some parts of Brazil, cultural festivities are celebrated in June. During this period, the incidence of firework-related ocular trauma increases, as observed in our study and supported by a previous study^(7)^. Interestingly, in the present study, a peak of cases was also observed in January, which was most likely to be related to the New Year’s celebrations.

Despite being a preventable ocular trauma, fireworks remain a relevant cause of ocular trauma, sometimes leading to irreversible visual impairment and/or blindness^(1)^. Although the human eye has natural protection barriers and protection gears are commercially available to prevent ocular injuries, accidents and traumatisms still contribute significantly to monocular and binocular blindness worldwide^(1)^. In addition, given that ocular injuries are more likely to affect young individuals, it results in medical leaves and even permanent disabilities, which has significant social and economic effects. The World Health Organization highlights for decades the need for regulatory measures involving the manufacturing and use of fireworks^(27)^, whereas the American Academy of Pediatrics suggests that its private use and by unqualified individuals should be banned^(28)^. Their concern is well justified, considering that numerous accidents involve children because of misuse or failure of the devices and absence of adult supervision^(7,29)^.

This study brings awareness to worse visual prognosis among firework victims who were injured in the countryside and outside the state of Pernambuco. This finding may alert authorities about the reduced number of ophthalmological facilities in remote areas and possible physical barriers to obtain healthcare and medical assistance, which we can consider with caution as causes of worse prognosis. In addition, this study revealed that firework-related ocular traumas are most seen during festive months (January and June) in Brazil and they affect more men in economically active age. Moreover, approximately one-quarter of patients needed surgical intervention. Thus, given the social and economic burden of firework-related ocular trauma, Brazilian authorities must work on prevention measures such as providing information to the population about its risks and its conscious use during cultural events and regulate personal protective equipment to reduce its incidence.
